# Mediation of Self-Compassion on Pathways from Stress to Psychopathologies among Japanese Workers

**DOI:** 10.3390/ijerph191912423

**Published:** 2022-09-29

**Authors:** Yasuhiro Kotera, Holly Young, Sarah Maybury, Muhammad Aledeh

**Affiliations:** 1 School of Health Sciences, University of Nottingham, Nottingham NG7 2HA, UK; 2 College of Health, Psychology and Social Care, University of Derby, Derby DE22 1GB, UK

**Keywords:** self-compassion, stress, psychopathology, mental health, Japanese workers, mediation

## Abstract

As awareness of mental health increases worldwide, how to improve mental health has begun to be discussed in many countries. Stress is known to cause diverse physical and mental health problems, including psychopathologies. On the other hand, our previous studies identified that self-compassion, kindness and understanding towards oneself are key components for good mental health in many populations, including Japanese workers. The government reports that Japanese workers suffer from high rates of mental health problems. However, the mechanism of how self-compassion helps their mental health remains to be evaluated. Accordingly, this study aimed to elucidate how self-compassion intervenes in pathways from stress to psychopathologies, namely depression and anxiety. One hundred and sixty-five Japanese workers completed an online survey regarding self-compassion, depression, anxiety and stress. Correlation and path analyses were conducted. These four variables were significantly inter-related. While self-compassion mediated the pathway from stress to depression, it did not mediate the pathway from stress to anxiety. These exploratory insights assist in understanding the mechanism of how self-compassion improves mental health and inform effective methods to implement self-compassion interventions to the Japanese workforce.

## 1. Introduction

### 1.1. Poor Mental Health among Japanese Workers

Stress damages our ability to cope with life’s challenges and causes problems with social functioning [1], leading to diverse mental health problems including depression and anxiety [2]. In Japan, a high number of workers report mental health issues, with over 60% of employees reporting intense depression and anxiety [3].

The number of Japanese workers diagnosed as depressed has increased steadily year on year over the past two decades [4]. There was a seven-fold increase in the number of compensation-claims for mental health problems within the Japanese workforce between 2000 and 2015 [1], and half of all suicides of the employed in Japan in 2015 were due to work-related causes [2]. During the second wave of the COVID-19 pandemic, April–May 2020, one-third of Japanese healthcare workers reported experiencing burnout [3]. At the time of writing, late-August to early September 2022, Japan is undergoing the seventh wave, and the mental health difficulties are expected to continue [4]. Research has shown that Japanese employees who experience poor communication with colleagues and supervisors, high levels of job overload and low levels of job satisfaction are at high risk of depression [5].

At work, the effects of poor mental health can include poorer performance and achievement [6], lost productivity, absenteeism, presenteeism, and substance abuse [7]. Subsequently, mental health at work is recognised as a pressing global issue, with the United Nations and many developed countries implementing responses to this challenge [8,9]. The problem is also seen as a national responsibility in Japan [10]. The government has addressed the issue over the past two decades by implementing initiatives focusing on occupational health consultations and medical examinations [11] as well as the work-style reform act to reduce overtime working [12] and karoshi (i.e., death from overwork) [13]. However, 40% of companies in Japan have not engaged with the government’s work-style reform and are unconvinced of its efficacy [14]. Japanese workers operate in a collectivistic and success-driven society, and attitudes towards poor mental health are negative [12]. Research has shown that Japanese workers believe poor mental health to be the result of such factors as institutionalism and weak personality [15]. Furthermore, many Japanese people (43%) report that they would be ashamed to seek help for mental health problems [16], placing them at higher risk of developing psychopathologies [17].

### 1.2. High Stress as the Cause of Psychopathologies

The links between stress, depression and anxiety are well established [18]. Though there are several established definitions [19], one common definition of psychological stress is a physical, mental or emotional factor that causes bodily or mental tension, and that requires behavioural adjustment [20,21]. Stress is a normal part of human existence but has become of worldwide concern due to the prevalence of its chronic form and its links with a wide range of health problems, from cardiovascular disease to suicide [22]. It is a highly individual phenomena, contingent on personal vulnerabilities and levels of resilience [23] and is conceptualised as a key risk factor for individuals in the development of depression and anxiety [24]. A considerable body of research has demonstrated that, prior to major depressive episodes, stressors are present at a significantly higher level than is usual, both in clinical and community samples [25]. That most depressive episodes are preceded by stressful life events has been borne out of recent research into responses to the COVID-19 pandemic, which has found high prevalence of anxiety and depression in healthcare workers operating at the frontline of the crisis [26,27]. Work-related stress is often a major source of psychological burden for individuals [28] and in Japan is the leading cause of stress, ahead of other domains including health, finances and family [29]. Although work-related stress can have multiple causes, it is normally conceptualised as arising when employees feel they have excessive demands placed on them which outweigh their abilities and knowledge, often with accompanying feelings of minimal control over work processes and little support from colleagues and supervisors [30,31].

Although depression and anxiety demonstrate high diagnostic comorbidity and are strongly correlated in both clinical and non-clinical self-report samples, they are conceptually distinct constructs [32,33]. Depression is characterised by feelings of sadness, emptiness, irritability, hopelessness, emotional and motivational deficits [34], and is often linked to problems with relationships, work, physical health and suicidal behaviour [35]. Anxiety is distinguished by excessive worry and fear and associated behavioural disturbances [34]. It can be seen as the emotional response to the appraisal of threat [36] and is to some extent characterised by a focus on apprehension as a stimulus [37]. Anxiety disorders are wide ranging and encompass phobias and panic disorders [38]. Stress is a risk factor for depression and anxiety, which are two different constructs. 

### 1.3. Self-Compassion Protects Mental Health

Self-compassion is defined as kindness and understanding towards oneself in difficult times [39]. Self-compassion has been identified as a protective factor for mental health in many populations [40,41,42], including Japanese workers [17,43]. Self-compassion changes an individual’s relationship with their problems, thereby reducing their psychological distress. It does this by fostering a healthy relationship with and attitude towards the self [44]; by being kind to oneself rather than judgmental, being mindful of painful thoughts rather than overidentifying with them, and by viewing experiences as part of the human condition rather than in isolation [45,46]. Self-compassion interventions have been successful in many populations, producing significant improvements in psychosocial outcomes in multiple randomised controlled trials [47] and in alleviating depression and anxiety in community samples [48,49,50]. Furthermore, self-compassion interventions have been shown to be more effective when administered to groups over individuals [47] and could therefore be a valuable and economical tool for use with workplace populations. A systematic review identified a negative association between work-related stress and self-compassion: employees who are kind towards themselves in difficult situations tend to feel less stress from work [49]. Moreover, organisational training that targeted self-compassion improved employee wellbeing through reduction in work stress [51,52]. These findings highlight a strong link between self-compassion and stress.

How self-compassion engages with the stress–psychopathology pathway remains to be evaluated, however. Having compassion for oneself would imply that individuals may act to prevent the experience of suffering via proactive wellbeing behaviours, potentially preventing stress from developing into psychopathologies [53,54]. Samples suffering from anxiety and depression, but not stress, have reported finding self-compassion of use in managing these psychopathologies, as it is a meaningful concept to them [55]. However, depression and anxiety were also reported to have a potentially negative impact on the ability to be self-compassionate via different mechanisms: depression was linked to self-loathing which was seen as a barrier to self-compassion, whilst an inability to think logically due to a focus on the source of anxiety was seen to inhibit self-compassionate thought processes [55,56]. It is clear from the evidence that self-compassion helps with mental health, but how or why this occurs in the stress–psychopathologies relationship has not been established. A compassion theory model, the three emotion regulatory systems, posits that compassion activates our soothing emotion system, which is related to psychological safety, reassurance, and calmness, leading to good mental health [57]. When we encounter stressful situations, we perceive stress (though the level of perceived stress depends on the individual’s coping skills such as cognitive reappraisal [58]), and that can result in depression and/or anxiety. It is possible that self-compassion mediates these pathways (namely stress–depression, and stress–anxiety pathways) by activating our soothing system. However, differences in these constructs (i.e., depression and anxiety) may affect the way in which self-compassion operates or involve different mechanisms altogether. These relationships have not been evaluated in Japanese workers. To explore these relationships, we used path analysis to examine whether self-compassion mediates the pathway from stress to these two distinct but overlapping psychopathologies.

### 1.4. Study Aims

This study had two aims. First, the study aimed to evaluate the relationships among self-compassion, depression, anxiety, and stress (Aim 1). Second, the study aimed to examine whether self-compassion mediates the pathways from stress to psychopathologies, namely depression and anxiety (Aim 2). Two hypotheses were established:

**H1:**  *Self-compassion mediates the pathway from stress to depression*.

**H2:**  *Self-compassion mediates the pathway from stress to anxiety*.

## 2. Materials and Methods

### 2.1. Participants

To be eligible, all participants recruited for the current study had to be 18 years and above and be working at least three days a week at an organisation in Japan. Out of 201 Japanese workers who agreed to take part in this study, a convenience sample of 165 Japanese workers, of which were 125 males and 40 females (Age M = 47.20, SD = 11.85, Range 20–73 years), 155 full-time workers, and 10 part-time workers completed a self-reported measure about their mental health condition using self-compassion, depression, anxiety, and stress. Ethical approval was granted by the University of Amsterdam Research Ethics Committee (Ref: 2019-WOP-10266).

In this workers’ sample, 7.3% of participants worked in education and construction, respectively (*n* = 12), 6.7% worked in retail, communication, and technologies, respectively (*n* = 11), 4.9% worked in wholesale and transit, respectively (*n* = 8), while the remaining workers worked in finance, manufacturing, hospitality, and caring industries. Most of these workers (66%) had a university degree as their highest qualification (*n* = 110), 17% had an advanced degree (*n* = 28), 15% had a high school diploma (*n* = 25) and 1% a middle school diploma (*n* = 2). In comparison with the general working population, there were more males in our sample (33 million male workers vs. 26 million female workers in Japan [59]); however, our sample mean age was similar (43 years old [60]). The number of participants satisfied the required sample size of 84 based on statistical power calculations (two tails, *p* H1 (*r*) = 0.30, *α* = 0.05, Power = 0.80, *p* H0 = 0 [61]). There was no compensation for participation. The study followed the Strengthening the Reporting of Observational Studies in Epidemiology reporting guidelines [62].

### 2.2. Procedure

Once the consent form was submitted, participants received a link to the online self-report scales. Before the analyses, data were screened for outliers and distribution. First, correlation analyses were used to appraise the relationships between self-compassion, depression, anxiety, and stress (Aim 1). Second, path analyses were conducted to elucidate whether self-compassion mediates pathways from stress to depression, and stress to anxiety (Aim 2). SPSS 27.0 (IBM, Armonk, NY, USA) and Process Macro (Columbus, OH, USA) were used [63].

### 2.3. Measures

Two self-report measures were used to assess the levels of depression, anxiety, stress, and self-compassion.

The Depression Anxiety and Stress Scale 21 (DASS-21) examined depression, anxiety and stress experienced in daily life. This 21-item scale is a briefer version of the DASS-42 [64]. The 21 items are organised into 3 subscales (seven items each): depression (e.g., ‘I couldn’t seem to experience any positive feeling at all’), anxiety (e.g., ‘I felt I was close to panic’), and stress (e.g., ‘I found it hard to wind down’). Each item is responded to on a four-point Likert scale (0 = ‘Did not apply to me at all’ to 3 = ‘Applied to me very much, or most of the time’). All subscales in the DASS-21 have good reliability (*α* = 0.87–0.94) [65].

Self-compassion was evaluated with the Self-Compassion Scale-Short Form (SCS-SF) [66]. SCS-SF is a 12-item version of the original 26-item Self-Compassion Scale [67]. The SCS-SF identifies to what degree one can be kind and understanding towards themselves when facing difficulties [67]. A five-point Likert scale (1 = ‘Almost never’ to 5 = ‘Almost always’) is used to respond to the 12 items (e.g., ‘When I feel inadequate in some way, I try to remind myself that feelings of inadequacy are shared by most people.’). The SCS-SF has good reliability (*α* ≥ 0.86 in all tested samples) [66].

## 3. Results

No outliers were identified. All skewness and kurtosis values indicated normal distribution (skewness −2 to 2, kurtosis −7 to 7 [68]). All variables demonstrated high internal consistency (*α* > 0.70) (Table 1).

### 3.1. Relationships, Self-Compassion, Depression, Anxiety and Stress (Aim 1)

Pearson correlations were used to evaluate the relationships among self-compassion, depression, anxiety and stress in Japanese workers (Table 1). For gender, point biserial correlations were calculated (1 = male, 2 = female).

Self-compassion was negatively associated with depression, anxiety and stress. Depression, anxiety and stress were positively inter-related. Age was associated with female gender and negatively associated with anxiety.

### 3.2. Mediation of Self-Compassion for Stress to Psychopathologies (Aim 2)

Two sets of path analyses were performed to appraise whether self-compassion mediates pathways from stress to psychopathologies, namely depression and anxiety. Model 4 in the Process macro [63] with 5000 bootstrapping re-samples and bias-corrected 95% confidence intervals (CIs) for indirect effects was used.

#### 3.2.1. Stress–Depression Pathway

**Figure 1:**
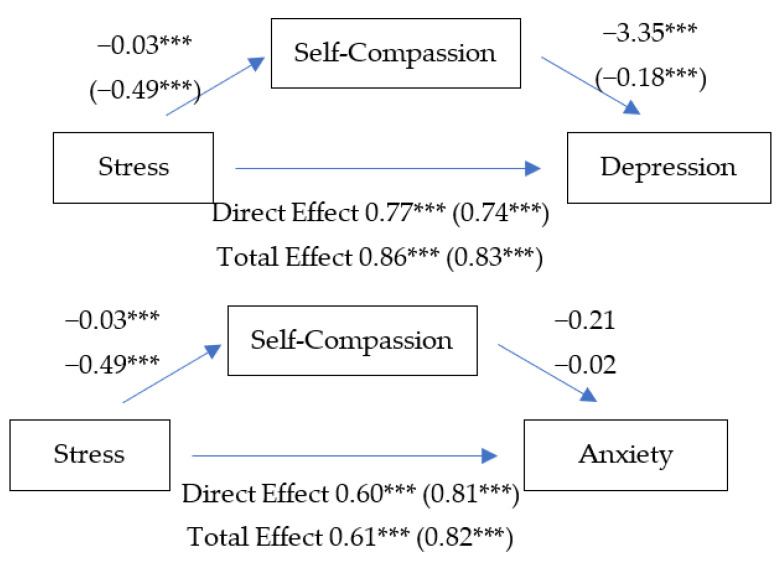
Parallel Mediation Model: Self-Compassion mediated the Stress–Depression Pathway but did not mediate the Stress–Anxiety Pathway. *** *p* < 0.001 (standardised coefficients).

Self-compassion partially mediated the pathway from stress to depression as all pathways were significant (Figure 1): from stress to depression (Direct effects *b* = 0.77, *p* < 0.001, BCa CI [0.67, 0.87]; Total effects *b* = 0.86, *p* < 0.001, BCa CI [0.77, 0.95]); from stress to self-compassion (*b* = −0.03, *p* < 0.001, BCa CI [−0.03, −0.02]); and from self-compassion to depression (*b* = −3.35, *p* < 0.001, BCa CI [−5.17, −1.52]). The indirect effect of stress on depression through self-compassion was also significant (*b* = 0.09, BCa CI [0.02, 0.17]). H1 was supported.

#### 3.2.2. Stress–Anxiety Pathway

Self-compassion did not mediate the pathway from stress to anxiety (Figure 1). Although pathways from stress to self-compassion (*b* = −0.03, *p* < 0.001, BCa CI [−0.03, −0.02]), from stress to anxiety (Direct effects *b* = 0.60, *p* < 0.001, BCa CI [0.53, 0.68]; Total effects *b* = 0.61, *p* < 0.001, BCa CI [0.54, 0.67]) were significant, a pathway from self-compassion to anxiety was not significant (*b* = −0.21, *p* = 0.76, BCa CI [−1.58, −1.16]). H2 was not supported.

## 4. Discussion

The mental health of Japanese workers has continued to worsen over the past 20 years, necessitating research into effective interventions to combat this growing trend. Self-compassion, or being kind to oneself in the face of personal hardship and negative events, has been shown to promote psychological wellbeing in Japanese workers [27]. While previous studies have demonstrated the value of self-compassion in promoting psychological wellbeing [69], more research is needed to better understand the mechanisms underpinning this relationship, which the current study aimed to do. Specifically, this study evaluated the relationships between the stress–psychopathology pathway and self-compassion, assessing whether self-compassion mediated pathways to depression and anxiety among Japanese workers. Self-compassion partially mediated the pathway from stress to depression (H1 supported), whereas it did not the pathway from stress to anxiety (H2 not supported).

Self-compassion was found to be strongly associated with stress and psychopathology, consistent with existing studies [70,71,72]. A study investigating self-compassion as a protective factor against stress in adolescents, highlighted that participants with enhanced self-compassion reported less perceived stress, as well as a reduced physiological stress response when faced with stressful social situations [73]. Higher levels of self-compassion can provide a shield against mental health issues, as this impacts an individual’s coping strategy when dealing with stressful life events [74,75,76]. Self-compassion is associated with a reduction in catastrophising when faced with challenges, and therefore can protect against the occurrence of more acute and lasting periods of distress such as in chronic depression [77].

Studies investigating this relationship specific to Japanese samples garnered comparable results. After undergoing an enhancing self-compassion program (ESP) to target poor mental health, Japanese participants reported a strengthened degree of self-compassion after treatment was complete, as well as a significant reduction in negative thoughts and emotions [78]. A comparative study between Japanese and Dutch workers found that self-compassion for Japanese workers negatively predicted mental health problems, suggesting that increased levels of self-compassion may bring improvements to their mental wellbeing [12].

In our sample, the stress–depression pathway was mediated by self-compassion, however the same effect was not present in anxiety. This suggests that when stress impacts depression, the level of self-compassion affects this pathway, whereas this does not occur in anxiety. These results are contrary to the past studies that have shown self-compassion to be a mediator of anxiety [69,79]. The difference found in the current study may come down to the different nature of anxiety and depression, namely the cognitive content specificity of each condition [80]. The cognitive content specificity hypothesis argues that specific features differentiate psychological disorders both at a symptomatic and diagnostic level [81]. Anxiety disorders are associated with a magnified sense of threat and danger, versus depression, which can result in a negative lens of oneself, one’s surroundings and prospects [82]. Furthermore, as suggested by Hong [83], there are distinct cognitive processes underlying their symptoms. Anxiety is associated with worry, thought patterns aimed at solving uncertain events to obtain a sense of control [75]. Conversely, depression is associated with rumination, the persistent focus on one’s negative emotions, past events and potential negative future outcomes [75]. Both worry and rumination possess similarities and can co-occur in anxiety and depression [84]; however, worry has been found to be a unique predictor of symptoms associated with anxiety [85]. While these distinctions between anxiety and depression have been challenged [86] when considered in relation to the tenets of self-compassion, they may have some explanatory power for the findings of the current study. Self-compassionate individuals are more likely to employ adaptive emotional regulation techniques, whereby negative situations are perceived as temporary and manageable [87]. This works to engender resilience and counter the development of depressive symptoms specifically, but not necessarily anxiety symptoms, by reducing the tendency towards rumination and experiential avoidance [88].

Based on these findings, an intervention focused on self-compassion should target depression more than anxiety in employees [89,90,91,92]. Compassion-focused therapy may be used to reduce the self-criticism and shame commonly experienced by individuals with depression, to shift the focus on fostering kindness and acceptance of oneself, and sense of being connected to others [93]. Acceptance and commitment therapy (ACT) may also be effective, incorporating elements of mindfulness to reduce self-judgment, feelings of isolation, and direct their focus to the individual they would like to be instead of punishing themselves for their perceived shortcomings [79]. In a study investigating the mental health of Swedish social workers, three 3 h sessions of ACT, with a specific focus on stress management, led to a significant decrease in stress and burnout [94]. Additionally, mindfulness training has been shown not only to reduce work-related stress, but also enhance workplace engagement and peer relationships [95]. Considering the continued challenges with work mental health in COVID-19, these insights may be especially helpful to the Japanese workforce and those who work with them. During the pandemic, in addition to stress, loneliness was highlighted as salient among workers in Japan [31]. A recent meta-analysis reported, though the study quality needs to be improved, these compassion- or mindfulness-based interventions are effective for reducing loneliness [96]. These compassion and mindfulness interventions are recommended for Japanese workers today.

A helpful intervention for workers experiencing anxiety could include forest bathing (shinrin-yoku), whereby individuals surround themselves by nature, usually by walking in a forest and practicing mindfulness [22,97,98]. A therapy programme originating in Japan, forest bathing is a familiar practice in the country, and has been found to be especially effective in reducing anxiety levels in the Japanese workforce [99]. Moreover, in a society where poor mental health is associated with shame and stigma [17], it may be easier to implement a local practice to address mental health needs. As mental health continues to be a global concern, forest bathing provides a cost-effective and accessible method to address the needs of individuals facing various mental health disorders in Japan and beyond, with studies demonstrating its effectiveness in Taiwan [100], Italy [101], and Poland [102]. In the Polish example, a short, one-day forest recreation, which consisted of observing, listening to, and touching elements of the forest saw an increase in the physiological and psychological wellbeing in students and young workers [90]. This traditional Japanese approach can be used for today’s Japanese employees.

Limitations of this study need to be noted. First, our sample was male oriented and consisted of highly educated people, workers from various industries, and a mixture of full-time and part-time workers; therefore, generalisability of our findings needs further evaluation. Indeed, whether these differences are associated with mental health difference remains to be evaluated [103,104]. Second, our recruitment only focused on participants who were comfortable using an online survey; therefore, we missed those who did not feel comfortable using this form of survey. Third, we used self-report scales; thus, response biases might have been present [105]. Relatedly, some of the used scales are being debated for their accuracy (e.g., SCS-SF [106]). Fourth, our study employed cross-sectional design; therefore, causality of these associations was not evaluated.

## 5. Conclusions

We found that self-compassion was indeed negatively associated with stress and psychopathology in Japanese workers. Moreover, when stress impacted depression, the level of self-compassion was important, whereas when stress impacted anxiety, a significant impact was not found with self-compassion. Our findings can help managers, HR staff and organisational psychologists to identify an effective way to introduce self-compassion to workplaces to protect employee mental health.

## Figures and Tables

**Table 1:** Correlations among self-compassion, depression, anxiety and stress in Japanese workers.

		M	SD	Skewness	Kurtosis	*α*	1	2	3	4	5	6
1	Age	47.20	11.85			-	-					
2	Gender (1 = M, 2 = F)	M(125), F(40)					−0.34 **	-				
3	Self-Compassion	3.02	0.49	−0.80	2.88	0.77	0.08	-0.01	-			
4	Depression	8.48	9.28	1.06	0.57	0.93	−0.14	0.03	−0.54 **	-		
5	Anxiety	4.99	6.59	1.64	2.55	0.87	−0.22 **	−0.02	−0.42 **	0.71 **	-	
6	Stress	8.81	8.87	0.92	−0.06	0.90	-0.14	0.05	−0.49 **	0.83 **	0.82 **	-

** *p* < 0.01.

## Data Availability

The data presented in this study are available on request from the corresponding author. The data are not publicly available due to ethical restrictions.
